# Social exclusion, infant behavior, social isolation, and maternal expectations independently predict maternal depressive symptoms

**DOI:** 10.1002/brb3.107

**Published:** 2012-11-29

**Authors:** John Eastwood, Bin Jalaludin, Lynn Kemp, Hai Phung, Bryanne Barnett, Jacinta Tobin

**Affiliations:** 1 School of Women's and Children's Health, University of New South WalesSydney, New South Wales, Australia; 2 School of Public Health and Community Medicine, University of New South WalesAustralia; 3 Centre for Research Evidence Management and Surveillance, South Western Sydney Local Health DistrictLiverpool, New South Wales, Australia; 4 Centre for Health Equity Training Research and Evaluation, University of New South WalesLiverpool, New South Wales, Australia; 5 Simpson Centre, South Western Sydney Area Health ServiceLiverpool, New South Wales, Australia; 6 School of Psychiatry, University of New South WalesSydney, New South Wales, Australia; 7 University of MelbourneMelbourne, Victoria, Australia

**Keywords:** Immigrants, postpartum depression, social isolation, social support, temperament

## Abstract

The objective of the study was to identify latent variables that can be used to inform theoretical models of perinatal influences on postnatal depressed mood and maternal–infant attachment. A routine survey of mothers with newborn infants was commenced in South Western Sydney in 2000. The survey included the Edinburgh Postnatal Depression Scale (EPDS) and 46 psychosocial and health-related variables. Mothers (*n* = 15,389) delivering in 2002 and 2003 were surveyed at 2–3 weeks for depressive symptoms. Nonlinear principal components analysis was undertaken to identify dimensions that might represent latent variables. Correlations between latent variables and EPDS >12 were assessed by logistic regression. A five-dimension solution was identified, which accounted for 51% of the variance among the items studied. The five dimensions identified were maternal responsiveness, social exclusion, infant behavior, migrant social isolation, and family size. In addition, the variable maternal expectation contributed significantly to total variance and was included in the regression analysis. Regression on EPDS >12 was predictive for all variables except for maternal responsiveness, which was considered an outcome variable. The findings are consistent with the proposition that social exclusion, infant behavior, social isolation among migrant mothers, and maternal expectations are determinants of maternal mood.

## Introduction

The early years are now known to play an important role in the genesis of adult health and disease. Current theory development seeks to explain complex perinatal mechanisms influencing the developmental origin of health and disease. The early psychosocial experiences of mothers and infants are of special significance to the development of secure mother–infant attachment with its positive impact on cognitive, emotional, social, and behavioral development. The aim of the study reported here is to identify latent variables that can be used to inform the building of theoretical models of perinatal influences on postnatal depression and maternal–infant attachment.

Postnatal depression has been estimated to affect 13–20% of women in studies carried out in industrialized nations (Gavin et al. 2005). Similar rates have also been found in large Australian studies (Buist et al. 2008; Eastwood et al. 2011). Psychosocial risk factors that have been implicated include history of mental illness, lack of social support, recent life stresses, personality variables, and negative feelings about pregnancy or parenthood (Austin and Priest 2005; Barnett et al. 2005). Beck, in her 2001 meta-analysis of 84 published studies, identified 13 significant predictors of postnatal depression. They were prenatal depression, self esteem, childcare stress, prenatal anxiety, life stress, social support, marital relationship, history of previous depression, infant temperament, maternity blues, marital status, socioeconomic status, and unplanned/unwanted pregnancy (Beck 2001).

The effect of maternal depressed mood on child development has been extensively studied. A meta-analysis of 46 observational studies of depressed mothers demonstrated a moderate association of maternal depression with negative (i.e., hostile, coercive) parenting behaviors and disengaged parenting behaviors (Lovejoy et al. 2000). The effects were strongest for studies of disadvantaged women.

Maternal postnatal depression has also been shown to be associated with impairment of cognitive development and secure attachment (Cogill et al. 1986; Murray et al. 1996). A meta-analysis of the relationship between maternal mental health and infant attachment, encompassing 35 studies and over 200 mother–infant pairs, indicated that maternal stress and depression are associated with a greater prevalence of insecure infant attachment (Atkinson et al. 2002).

Martins and Gaffan (2000) propose that attachment may be one, of several, pathways by which maternal depression causes later childhood problems. In their meta-analysis of seven studies, they found that infants of depressed mothers “showed significantly reduced likelihood of secure attachment and marginally raised likelihood of avoidant and disorganized attachment” (Martins and Gaffan 2000).

South Western Sydney is an area of substantial social disadvantage and a diverse multicultural population. Commencing in the late 1990s, the Mother and Infant Network (MINET) Program developed and implemented an integrated clinical data network, which included the routine interview of new mothers using a 45-item clinical and self-report survey known as the Ingleburn Baby Information Survey (IBIS) (Phung et al. 2001). The IBIS questionnaire includes administration of the Edinburgh Postnatal Depression Scale (EPDS) as a measure of maternal depressive symptoms (Cox et al. 1987). The scale indicates significant anxiety and depressive symptoms, but is not diagnostic. A score >12 indicates the probability of a formal diagnosis in an English-speaking population.

The nonlinear principal components analysis reported here is part of a multilevel and mixed-method exploration of factors that might be associated with postnatal depression and adversity and their possible impact on developmental outcomes of the infant. We have elected to use nonlinear principal component analysis (PCA) to identify dimensions in the data that may represent underlying latent (unmeasured) variables. The information thus gained will be used to inform the development theoretical models of perinatal influences on postnatal depression and maternal–infant attachment.

## Methods

### Study setting

The setting is the Local Government Areas of Bankstown, Fairfield, Liverpool, Campbelltown, Camden, Wollondilly, and Wingecarribee, in New South Wales (NSW), Australia. This area has a diverse multicultural population with 28.4% of the population having been born overseas compared with 17.8% for the rest of NSW. Twenty percent of infants are born to women from South-East, North-East, or Southern Asia. South Western Sydney is an area of substantial social disadvantage, and has lower education attainment and lower income levels than other parts of NSW.

### Study design

The study is a population-based cross-sectional study of mothers of infants born in South Western Sydney Area Health Service (SWSAHS) from 2002 to 2003. An exploratory data analysis approach was undertaken for the purpose of informing theory building (Behrens 1997). The exploratory data analysis included descriptive analysis of data, PCA and logistic regression. The results of the PCA and logistic regression, using the identified latent variables, are presented here. The 2002–2003 study (*n* = 15,389) is a subsample from a larger data set collected from 1998 to 2006. A 2004–2006 subsample was retained for subsequent confirmatory studies.

### Participants

The study utilized the IBIS database. This database was initiated in 1995 and is based on the routine survey by Child and Family Health Nurses of all mothers who attend the first well-baby clinic (home visit or clinic based) after discharge from the postnatal ward. The mean postpartum aged at interview was 3.77 weeks (95% CI 3.62–3.92).

Population-based collection started in Campbelltown and Wollondilly in 1998, followed by Bankstown in 2000, Fairfield and Wingecarribee in 2001, and Liverpool in 2002. The calendar years of 2002 and 2003 were used for this study as all geographical areas, and 92% of births (*n* = 2199) were surveyed. Of those surveyed, 70% percent consented to completing an EPDS and were included in this analysis. The mothers who did not complete an EPDS were more likely to report: difficult financial situation, public housing accommodation, low maternal education, not breast feeding, and short suburb duration.

### Variables

The IBIS survey contains 45 items, which are both clinical (e.g., weight, length, breastfeeding, hearing, and vision screening) and parental self-report in nature.

The variables selected for analysis were: mother's country of birth (Australia or other), Aboriginal or Torres Strait Islander culture, marital status, household size, blended family, number of children under 5 years of age, accommodation (privately owned or not), employment of mother, employment of father, financial situation (10-point scale), car access, phone access, mother's rating of her health (5-point scale), mother's rating of her child's health (5-point scale), breastfeeding (which included both exclusive and partial breastfeeding), smoking, mother's expectations (“Is being a mother what you expected” – 5-point scale), planned pregnancy, previous miscarriage, previous child death, previous stillbirth, previous child disability, previous termination of pregnancy, previous sudden infant death, suburb duration, regret about leaving the suburb (“If for some reason you had to leave this suburb would you be sorry to go?”), support network (“If you had any worries about your child, how many people do you feel you could turn to for help and support, not including health professionals?”), practical support (“Do you receive adequate practical support since the birth of the baby?”), emotional support (“Have you been able to talk to someone about how you are feeling since the birth of the baby?”), mother's response to her child (“Does the mother respond to the child's interactions of discomfort?”), mother comforts her child (“Does the mother show the ability to comfort the child?”), mother enjoys contact with the baby (“Does the mother enjoy close physical contact with the child?”), and “Since the birth of your baby how much time did your baby seem – to have trouble sleeping (5-point scale), to be a demanding baby (5-point scale), to be content (5-point scale), to be a difficult feeder (5-point scale), or to be difficult to comfort (5-point scale).”

As argued by Gorsuch (2003) not all variables available are required to be included in a factor analysis. The study dependent variable EPDS and variables from the clinical domain (i.e., infant weight, head circumference, length, hearing and vision screening, and referral type) were excluded in this analysis of psychosocial experiences.

### Statistical analysis

Factor analysis, and the related PCA approach, is based on a matrix of correlations between variables, and hence data assumptions for correlations and linear regression apply including the requirement for interval data that are normally distributed. The data in this study were categorical and contained a number of binary and nominal variables that might have nonlinear relationships with the ordinal Likert-scale variables. We therefore used nonlinear rather than linear analysis. As one of the goals was to construct composite variables for later modeling studies, we decided to use nonlinear PCA.

One of the new algorithmic models used for measuring latent variables is PCA with Optimal Scaling (Gifi 1990; Meulman et al. 2004), also known as categorical PCA (CatPCA). CatPCA is the nonlinear equivalent of PCA, but unlike PCA, CatPCA can manage categorical variables and does not require classical statistical assumptions, like multivariate normality.

CatPCA simultaneously reduces the dimensionality of the data and turns categorical variables into quantitative variables using optimal scaling. The quantitative measure obtained by CatPCA (object scores) takes into account the possible multidimensionality, the nature of variables, and their importance in determining the measure. The quantitative measures have coordinates that allow the categories or dimensions to be represented in a geometric display thus making data interpretation easier.

All variables in our data had integer values and it was not necessary, therefore, to discretize them for analysis. Missing values were treated passively, deleting persons with missing values only for those variables on which they had missing values. The following variables were treated as nominal: marital status, accommodation, employment of mother, employment of father, and education of mother. All other variables were treated as ordinal. With Likert scales with predominantly five categories, and the large sample size, we considered ordinal quantification to be appropriate.

To determine the adequate number of components to retain in the analysis, we generated a scree plot using the eigenvalues of the correlation matrix of the quantified variables from four-, five-, six-, and seven-dimensional solutions. Nonlinear PCA solutions are not nested, so a scree plot for a seven-dimensional solution – in which the sum of the seven largest eigenvalues is optimized – can be different from a scree plot for a five-dimensional solution, with the position of the elbow moving from the fifth to the fourth component. In the present analysis, different dimensionalities consistently placed the elbow at the fifth component.

Rotation is not available in the CatPCA software and for the nonlinear PCA solution on the IBIS data, rotation was not called for, as most variables already loaded highly on only one component.

The object scores obtained from the five dimensions were saved for subsequent analysis. Correlations between the scores and EPDS >12 were assessed by logistic-regression models with the exception of dimension 1 (maternal responsiveness), which was assessed using directed acyclic graphs to be an effect of the depressive symptoms measured by the EPDS. Odds ratios (ORs) were determined with 95% confidence intervals (95% CI). All analysis was undertaken using SPSS 19.0 © IBM 2010 (Armonk, New York).

### Ethics approval

The study obtained ethics approval from the Human Research Ethics Committee, South Western Sydney Area Health Service and from the University of NSW Human Research Ethics Committee.

## Results

Exploratory data analysis including nonlinear PCA solutions can best be interpreted through graphical visualization (Tukey 1980; Linting et al. 2007). The results section will focus on interpreting graphical outputs from CatPCA. The component loadings for a five-dimension analysis are shown in Table 1. Component loadings are arranged in decreasing order within dimensions, loadings greater than 0.40 are in bold and loadings less than 0.30 are suppressed to aid interpretation.

**Table 1:** Component loadings for five dimensions

	Dimension				
	
Variable	1	2	3	4	5
Responds to child	**1.148**	−.336			
Comfort child	**1.129**	−.316			
Enjoys contact	**1.103**	−.300			
Accommodation		**.560**	−.334	−.343	
Financial situation		**.528**			
Access to car		**.518**			
Employment of mother		**.512**			
Employment of father		**.455**			
Social support network		**.435**		**.424**	
Mothers health		**.421**		**.421**	
Marital status		**.413**	−.308	−**.562**	
Country of birth				**.527**	
Health of child		.400		**.492**	
Education of mother		.386			
Emotional support		.370			
Unplanned pregnancy		.363		−.344	
Practical support		.345			
Regret leaving suburb		.328			
Baby trouble sleeping			**.751**		
Baby difficult to comfort			**.741**		
Baby demanding		.320	**.735**		
Baby difficult feeder			**.612**		
Baby content			**.664**		
Number of children under 5					**.746**
Household size					**.634**
Suburb duration					
Maternal expectations			.301		
Breast feeding					
Total (Eigenvalue)	4.16	3.67	3.21	1.91	1.38
Cronbach's α	0.79	0.75	0.72	0.48	0.28
Percentage of variance	14	13	11.5	6.8	4.9

Component loadings greater than 0.40 are in bold and loadings less than 0.30 are suppressed.

### Variance

The five-dimensional nonlinear PCA yields an eigenvalue of 4.16 for the first component. This is approximately 14.8% of the variance of the transformed variables (4.16/28 with 28 being the number of variables). The eigenvalue of the second component is 3.67 (13%), third is 3.21 (11.5%), fourth is 1.91 (6.8%), and the fifth is 1.38 (4.9%). The total variance in the transformed variable accounted for by the five dimensions is 51%.

### Biplots of component loadings

**Figure 1:**
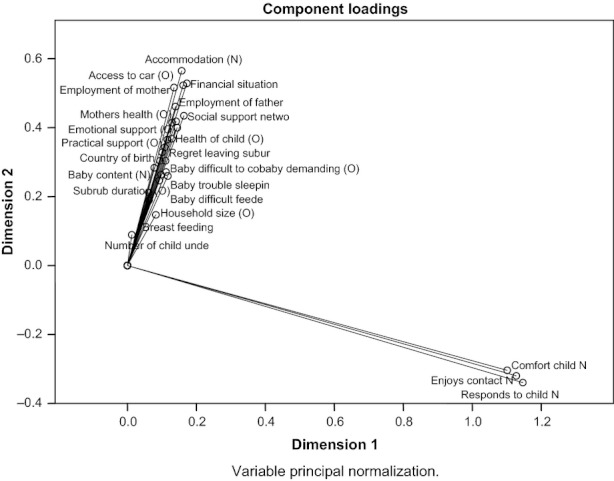
Biplot of dimensions 1 and 2.

**Figure 2:**
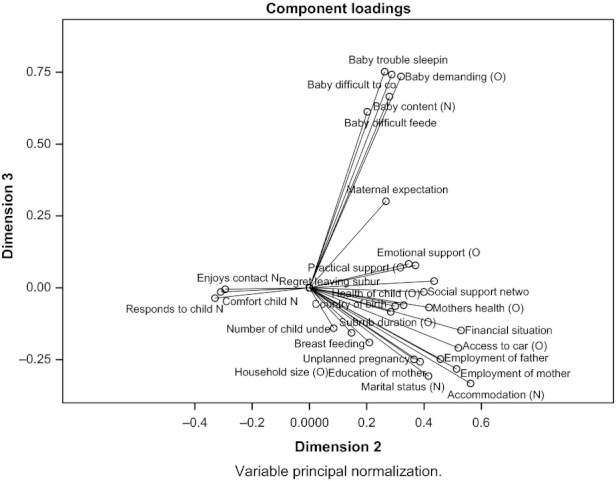
Biplot of dimensions 2 and 3.

**Figure 3:**
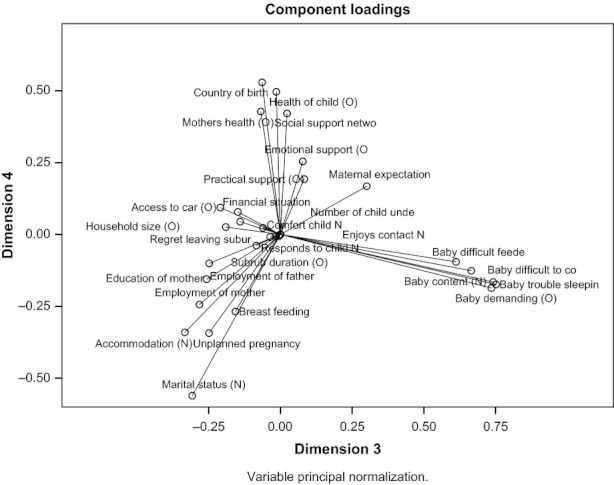
Biplot of dimensions 3 and 4.

Component loadings are presented in Figures 1–3 displayed as vectors. The component loadings range between −1 and 1, and indicate the Pearson correlations between the quantified variables and the principal components. The coordinates of the end point of each vector are the loadings of each variable on the two components plotted. Variable vectors that are close together in the plot are closely and positively related. Variables with vectors that make approximately a 180° angle with each other are closely and negatively related. Variables vectors with a 90° angle are not related (Linting et al. 2007).

The variables in Figure 1 form two clearly distinct groups. The vector lines are relatively long, indicating that the first two dimensions account for a large amount of the variance of all of the quantified variables.

Three variables form a bundle with a large positive loading on the first dimension. These variables (comforts child [1.129], enjoys contact [1.103], and responds to child [1.148]) may be considered representative of *maternal responsiveness* and bonding to the infant. The vectors in this bundle are orthogonal (perpendicular) to the other vectors, which indicate that this set of variables is uncorrelated with the second set of variables.

The second dimension includes all remaining variables with the largest loadings being for the variables: accommodation (0.560), employment of mother (0.512), access to a car (0.518), financial situation (0.528), marital status (0.413), education of mother (0.386), social support network (0.435), mother's health (0.421), and unplanned pregnancy (0.363). The second dimension might represent a latent variable related to *social exclusion*.

In Figure 2, the second dimension variable vectors for financial situation, access to car, employment of mother and father, accommodation, education status of mother, marital status, and unplanned pregnancy are closely correlated. This bundle of vectors may represent a common latent variable related to *social exclusion*.

The variables: baby content (0.664), baby trouble sleeping (0.751), baby demanding (0.735), baby difficult feeder (0.612), and baby difficult to comfort (0.741), form another bundle, which is orthogonal to the other vectors, with the exception of maternal expectation. The variables in dimension three might be considered related to *infant behavior* and temperament. The variable vector for *maternal expectation* is intermediate between the *infant behavior*–related variables and the variables for emotional, practical, and social support suggesting some correlation with those vectors. Also of note is the strong negative relationship between variables related to maternal attachment and social support network and no regret leaving the suburb.

In Figure 3, the variable vectors for country of birth (0.527), health of child (0.492), social support network (0.424), mother's health (0.421), form a bundle that is negatively correlated with marital status (−0.562), unplanned pregnancy (−0.344), public accommodation (−0.343), and not breastfeeding (−0.268). These vectors may represent *social isolation* among migrant mothers.

The fifth dimension, *family size* (not plotted), is predominantly composed of the variables number of children under 5 (0.746) and household size (0.634). There is a negative loading on suburb duration (−0.295) and no regret leaving the suburb (−0.230). This dimension is not strongly associated with variables in other dimensions.

### Regression analysis

Maternal responsiveness (dimension 1) was considered an outcome of maternal depressive symptoms and accordingly was not included in the multivariate model. The other four dimensions were all significantly associated with EPDS >12 in the univariate analysis (Table 2). Dimension 5 – *family size* was protective of depressive symptoms. The variable *maternal expectation* had a long vector in the biplots indicating that it accounted for a large amount of variance. It did not, however, load onto one of the five dimensions. Consequently, we elected to include *maternal expectation* in the regression studies. *Maternal expectation* was strongly predictive of EPDS >12 (OR 2.77; CI 95%: 2.55–3.01).

In the multivariate model *social exclusion*, *infant behavior*, *migrant isolation*, and *maternal expectation* remain significant. *Family size* (dimension 5) is no longer significant when controlling for the other dimensions and *maternal expectation* (Table 2). For the multivariate model, the Hosmer and Lemeshow Test was not significant (χ^2^ = 11.1, *df* 8, *P* = 0.169) indicating that the data fit the model well. The model was able to correctly classify 100% of EPDS >12 for an overall success rate of 92.4%. The Hosmer and Lemeshow Test for a model with dimension 5 – *family size* removed indicated a poorer fit.

**Table 2:** Univariate and multivariate logistic regression on EPDS >12

							95% CI for Exp(B)	
							
	B	SE	Wald	*df*	Sig.	Exp(B)	Lower	Upper
Univariate logistic regressions								
F2-Social exclusion	.732	.032	531.541	1	.000	2.079	1.954	2.212
F3-Infant behavior	.326	.029	125.575	1	.000	1.385	1.308	1.466
F4-Migrant isolation	.301	.034	79.949	1	.000	1.351	1.265	1.443
F5-Large household	−.122	.034	12.550	1	.000	.885	.828	.947
Maternal expectations	1.019	.042	576.893	1	.000	2.771	2.550	3.012
Multivariate logistic regression model								
F2-Social exclusion	.586	.035	285.278	1	.000	1.797	1.679	1.923
F3-Infant behavior	.127	.031	17.198	1	.000	1.135	1.069	1.205
F4-Migrant isolation	.206	.033	39.963	1	.000	1.229	1.153	1.310
F5-Large household	−.030	.033	.831	1	.362	.970	.909	1.036
Maternal expectations	.718	.047	228.817	1	.000	2.051	1.869	2.251
Constant	−5.085	.166	939.442	1	.000	.006		

## Discussion

In our survey of mothers of infants born in South West Sydney from 2002 to 2003, we identified a five-dimension solution using nonlinear PCA for ordinal, nominal, and dichotomous items. The solution accounted for 51% of the variance among the items studied. The five dimensions identified may represent important underlying latent variables that have causal relationships with maternal depressive symptoms. In addition to the five identified dimensions, the variable *maternal expectation* was identified as contributing significantly to total variance. *Maternal expectation* did not cluster with one of the five identified dimensions and has therefore been analyzed separately.

The first identified dimension, *maternal responsiv*eness included the three variables, *enjoys contact with the child*, *comforts the child*, and *responds to the child*. Interestingly, the vectors for this dimension were perpendicular to other vectors indicating that this dimension is uncorrelated to the other variables in the data set. Poor maternal responsiveness to the infant is recognized as an important outcome of maternal depressive symptoms.

The third identified dimension was *infant behavior*, which included: baby not content, -trouble sleeping, -demanding, -difficult feeder, and -difficult to comfort. Maternal depression has been shown to have an impact on infant behavior and attachment. Where a mother is depressed, the effects on her infant have been shown to be mediated by her “attachment state of mind” (McMahon et al. 2006). There has been less research on the impact of infant temperament on maternal stress and depression. Beck in her systematic review found that infant temperament was moderately related to postpartum depression (Beck 2001).

The role of the infant in the development of secure attachment is currently debated (Crockenberg and Leerkes 2000; Gervai 2009). One view is that the infant's temperament, in particular the intensity and pervasiveness of negative emotionality (i.e., irritability) is a primary determinant of attachment patterns. The other viewpoint emphasizes the dominant role of maternal sensitivity in determining the early infant–mother relationship. In this case, it is argued that difficult temperament can be accommodated by sensitive caregivers which can still foster secure attachment relationships. Fonagy et al. (1991) found that the infant attachment was predicted in over 70% of pairs by the parent's attachment state of mind as measured during pregnancy.

Gervai (2009) cites an extensive review by Vaugh and Bost (1999) as arguing “that temperament and attachment are separate constructs, [with] studies showing interrelationships on the one hand, and independence on the other result from different conceptualisations and assessments of both.” Gervai also draws attention to a body of empirical research, which demonstrates relationships between attachment quality and infant irritability, proneness to distress and stress regulation.

Mangelsdorf and Frosch (1999) have suggested that effects of infant temperament on attachment may be indirect and moderated by other maternal and social variables. This view is consistent with both viewpoints with infant temperament influencing attachment under certain maternal and social conditions.

The second identified dimension, *social exclusion* includes poor accommodation, unemployment of mother, no access to a car, difficult financial situation, single marital status, low education of mother, small social support network, poor self-reported mother's health, and unplanned pregnancy. Women of low socioeconomic status have consistently been found to have higher rates of antenatal and postnatal depression (O'Hara and Swain 1996; Beck 2001). The latent dimension identified is a broader concept than low socioeconomic status and includes elements of isolation and exclusion from society. The definition of social exclusion remains contested, but there is a common “understanding that social exclusion is not only about material poverty and lack of material resources, but also about the processes by which some individuals and groups become marginalised in society” (Millar 2007). A consensus definition proposed by Tsakloglou and Papadopoulos (2002) included measures of income poverty, living conditions, necessities of life, and social relations. The measures of social relations included meeting friends, talking to neighbors, and membership of clubs or groups. Saunders (2003) undertook a study of social exclusion in Australia and found that sole parents were the most excluded group. Saunders also found that lack of social interaction was the major form of social exclusion in the Australian setting, which is consistent with findings from this study.

The fourth identified dimension, *migrant social isolation* included: mother not born in Australia, poor social support network, poor self-reported health, and poor reported health of the child. Postnatal depression has been found to be more common among recent migrants to Australia (Williams and Carmichael 1985; Brown and Lumley 2000), Pacific Island mothers in Auckland, New Zealand (Abbott and Williams 2006), Canadian immigrants, asylum seekers and refugees (Stewart et al. 2008; Dennis et al. 2009), London ethnic minorities (Onozawa et al. 2003), and Latinas or Hispanic U.S. mothers (Beck et al. 2005; Diaz et al. 2007). The significance of these findings is complicated by the wide international cross-cultural variation definitions and understandings of postnatal depression and depressive symptoms. Halbreich and Karkun (2006) undertook a review of 143 studies from 40 countries and found a wide range in reported rates. The authors concluded that the variability might be due to cross-cultural variables, reporting style, differences in perception of mental health and its stigma, differences in socioeconomic environments and biological vulnerability factors. Of significance in our study is the possibility that migrant mothers are socially isolated or segregated within the South West Sydney community.

The fifth identified dimension, *family size*, was a complex combination of variables including number of children under five, household size and weak negative loading of suburb duration, and “no regret leaving the suburb.” Taken together, they represent mothers with larger families who have been in their suburb for some time and do not want to leave. It was not surprising, therefore that this dimension might protect mothers from depressive symptoms as it has been frequently reported that lack of social support is an important predictor of maternal depression (Beck 2001).

The vector for the variable *maternal expectations* was intermediate between the infant behavior-related variables and the variables for emotional, practical, and social support, suggesting some correlation with those vectors. The length of the vector suggested that the variable was important and independent from the identified latent variables. An association between maternal expectation's and depressive symptoms is consistent with previous studies (Beck 2002).

### Methodological limitations

The size (15,389) of this cross-sectional study of the EPDS administered to postnatal women is unique. The cross-sectional design, however, has limitations particularly in relation to drawing causal inferences from the regularities observed. Selection bias may have occurred from refusal and nonresponse in the study population. The self-report nature of the survey is particularly problematic with altered responses depending on mother's mental state.

There was a systematic nature to the missing EPDS data. Comparison of those who completed the EPDS and those who did not showed that statistically significant differences existed for financial situation, accommodation, maternal education, and breast feeding and suburb duration. Financial difficulties were more likely, for example, to be reported by mothers who did not have an EPDS. The direction of the bias is to reduce the strength of the association between financial difficulties and depressive symptoms.

This cross-sectional postnatal study is limited by the lack of prepregnancy and antenatal longitudinal data on depressive symptoms and related covariates. Future antenatal and postnatal data linkage will enable us to report longitudinal associations and incidence rates.

The study reported here has sought to identify latent variables using nonlinear PCA. The use of generated latent variables is contentious among epidemiologists who generally use empirically observed variables. Latent variables are more commonly used in the psychological and human development sciences to enable analysis of unobserved phenomenon such as intelligence and emotion. The use here of latent variables methods has enabled us to hypothesize about underlying unobserved phenomenon that may be causing postnatal depressive symptoms.

### Implication of findings

The five identified dimensions and the maternal expectations variable all had significant correlations with maternal depressive symptoms. The multiple regression analysis supported the proposition that social exclusion, infant behavior, social isolation among migrant mothers, and maternal expectations independently predict maternal depressive symptoms. They may also be directly, or indirectly, predictive of maternal responsiveness to her infant. Path analysis and structural modeling using a longitudinal data set will assist in confirming these propositions.

These findings have important implications for public health and migrant resettlement policies. The significant long-term consequences of postnatal depression and insecure attachment indicate that preventive interventions are warranted.

A recent comprehensive review, which included a number of sustained nurse home-visiting programs, found that the most promising intervention was the provision of intensive professional postpartum support (Dennis 2005). The efficacy of early nurse home visiting for postnatal depression has recently been confirmed (Morrell et al. 2009), but such programs are yet to be extended to all communities. In particular, few such programs exist in Australia for migrant families of non-English-speaking background. The role of antenatal groups in preventing postnatal depression has not yet been confirmed (Austin 2003). But, a recent study found that proactive telephone-based peer support was protective (Dennis et al. 2009). The study's findings related to maternal expectations have implications for antenatal education and counseling interventions. It may be beneficial to provide more information on the rewards and challenges of early parenthood (Harwood et al. 2007).

Difficult infant temperament is an important public health matter. Infant crying has a known association with physical abuse of infants by parents and other caregivers (Reijineveld et al. 2004). It is thus important that health services identify the importance of difficult infant temperament, in particular excessive infant crying, and make appropriate clinical interventions to prevent immediate harm and support the infant's long-term developmental trajectory.

The efficacy of early nurse home visiting for postnatal depression has recently been confirmed (Morrell et al. 2009). Such programmes are yet to be extended to all communities. The findings from this study suggest that priority should be given to providing support to mothers during pregnancy and after childbirth.

## References

[b1] Abbott MW, Williams MM (2006). Postnatal depressive symptoms among Pacific mothers in Auckland: prevalence and risk factors. Aust. NZ J. Psychiatry.

[b2] Atkinson J, Paglia A, Conne-Perreard E, Bousquet A, Manzano J (2002). Attachment security: a meta-analysis of maternal mental health correlates. Clin. Psychol. Rev.

[b3] Austin M (2003). Targeted group antenatal prevention of postnatal depression: a review. Acta Psychiatr. Scand.

[b4] Austin M-P, Priest SR (2005). Clinical issues in perinatal mental health: new developments in the detection and treatment of perinatal mood and anxiety disorders. Acta Psychiatr. Scand.

[b5] Barnett BE, Glossop P, Matthey S, Stewart H, Henshaw C, Elliott S (2005). Screening in the context of integrated perinatal care. Screening for perinatal depression.

[b6] Beck CT (2001). Predictors of postpartum depression: an update. Nurs. Res.

[b7] Beck CT (2002). Postnatal depression: a metasynthesis. Qual. Health Res.

[b8] Beck C, Froman R, Bernal H (2005). Acculturation level and postpartum depression in Hispanic mothers. MCN Am. J. Matern. Child Nurs.

[b9] Behrens J (1997). Principles and procedures of exploratory data analysis. Psychol. Methods.

[b10] Brown S, Lumley J (2000). Physical health problems after childbirth and maternal depression at six to seven months postpartum. BJOG.

[b11] Buist A, Austin M, Hayes BA, Speelman C, Bilszta J, Gemmill A (2008). Postnatal mental health of women giving birth in Australia 2002–2004: findings from the beyondblue National Postnatal Depression Program. Aust. NZ J. Psychiatry.

[b12] Cogill S, Caplan H, Alexandra H, Robson K, Kumar R (1986). Impact of maternal post-natal depression on cognitive development of young children. Br. Med. J.

[b13] Cox J, Holden J, Sagovsky R (1987). Detection of postnatal depression. Development of the 10-item Edinburgh Postnatal Depression Scale. Br. J. Psychiatry.

[b14] Crockenberg S, Leerkes E, Zeanah C (2000). Infant social and emotional development in family context. Handbook of infant mental health.

[b15] Dennis C-L (2005). Psychosocial and psychological interventions for prevention of postnatal depression: systematic review. Br. Med. J.

[b16] Dennis C, Hodnett E, Kenton L, Weston J, Zupancic J, Stewart D (2009). Effect of peer support on prevention of postnatal depression among high risk women: multisite randomised controlled trial. Br. Med. J.

[b17] Diaz M, Le H, Cooper B, Muñoz R (2007). Interpersonal factors and perinatal depressive symptomatology in a low-income Latina sample. Cultur. Divers. Ethnic Minor. Psychol.

[b18] Eastwood J, Phung H, Barnett B (2011). Postnatal depression and socio-demographic risk: factors associated with Edinburgh Depression Scale scores in a metropolitan area of New South Wales, Australia. Aust. NZ J. Psychiatry.

[b19] Fonagy P, Steele H, Steele M (1991). Maternal representations of attachment during pregnancy predict the organization of infant–mother attachment at one year of age. Child Dev.

[b20] Gavin N, Gaynes B, Lohr K, Meltzer-Brody S, Gartlehner G, Swinson T (2005). Perinatal depression: a systematic review of prevalence and incidence. Obstet. Gynecol.

[b21] Gervai J (2009). Environmental and genetic influences on early attachment. Child Adolesc. Psychiatry Ment. Health.

[b22] Gifi A (1990). Nonlinear multivariate analysis.

[b23] Gorsuch R, Schinka J, Velicer W (2003). Factor analysis. Handbook of psychology: research methods in psychology.

[b24] Halbreich U, Karkun S (2006). Cross-cultural and social diversity of prevalence of postpartum depression and depressive symptoms. J. Affect. Disord.

[b25] Harwood K, McLean N, Durkin K (2007). First-time mothers' expectations of parenthood: what happens when optimistic expectations are not matched by later experiences?. Dev. Psychol.

[b26] Linting M, Meulman J, Groenen P, van der Kooij A (2007). Nonlinear principal components analysis: introduction and application. Psychol. Methods.

[b27] Lovejoy C, Graczyk P, O'Hare E, Neuman G (2000). Maternal depression and parenting behaviour: a meta-analytic review. Clin. Psychol. Rev.

[b28] Mangelsdorf S, Frosch C (1999). Temperament and attachment: one construct or two?. Adv. Child Dev. Behav.

[b29] Martins C, Gaffan E (2000). Effects of early maternal depression on patterns of infant–mother attachment: a meta-analytic investigation. J. Child Psychol. Psychiatry.

[b30] McMahon CA, Barnett BE, Kowalenko N, Tennant C (2006). Maternal attachment state of mind moderates the impact of postnatal depression on infant attachment. J. Child Psychol. Psychiatry.

[b31] Meulman J, Heiser A, Van der Kooij W, Kaplan D (2004). Principal components analysis with nonlinear optimal scaling transformations for ordinal and nominal data. Handbook of quantitative methodology for the social sciences.

[b32] Millar J, Abrams D, Christian J, Gordon D (2007). Social exclusion and social policy research: defining exclusion. Multidisciplinary handbook of social exclusion research.

[b33] Morrell C, Warner R, Slade P, Dixon S, Walters S, Paley G (2009). Psychological interventions for postnatal depression: cluster randomised trial and economic evaluation. The PoNDER trial. Health Technol. Assess.

[b34] Murray L, Fiori-Cowley A, Hooper R, Cooper PJ (1996). The impact of postnatal depression and associated adversity on early mother-infant interaction and later infant outcome. Child Dev.

[b35] O'Hara M, Swain A (1996). Rates and risk of postnatal depression – a meta-analysis. Int. Rev. Psychiatry.

[b36] Onozawa K, Kumar R, Adams D, Dore C, Glover V (2003). High EPDS scores in women from ethnic minorities living in London. Arch. Womens Ment. Health.

[b37] Phung H, Young L, Greenfield D, Bauman A, Hillman K (2001). A framework for monitoring maternal and infant health status. Aust. Health Rev.

[b38] Reijineveld S, Brugman M, van der Wal E, Hira Sing R, Verloove-Vanhorick S (2004). Infant crying and abuse. Lancet.

[b39] Saunders P (2003). Can social exclusion provide a new framework for measuring poverty.

[b40] Stewart D, Gagnon A, Saucier J, Wahoush O, Dougherty G (2008). Postpartum depression symptoms in newcomers. Can. J. Psychiatry.

[b41] Tsakloglou P, Papadopoulos F (2002). Aggregate level and determining factors of social exclusion in twelve European countries. J. Eur. Soc. Policy.

[b42] Tukey J (1980). We need both exploratory and confirmatory. Am. Stat.

[b43] Vaugh B, Bost K, Cassidy J, Shaver P (1999). Attachment and temperament: redundant, independent, or interacting influences on interpersonal adaptation and personality development?. Handbook of attachment: theory, research and clinical applications.

[b44] Williams H, Carmichael A (1985). Depression in mothers in a multi-ethnic urban industrial municipality in Melbourne. Aetiological factors and effects on infants and preschool children. J. Child Psychol. Psychiatry.

