# Social exclusion, infant behavior, social isolation, and maternal expectations independently predict maternal depressive symptoms

**DOI:** 10.1002/brb3.140

**Published:** 2013-05-13

**Authors:** John E D Eastwood, Bin Jalaludin, Lynn Kemp, Hai Phung, Bryanne A M Barnett, Jacinta Tobin

*Brain and Behavior* 2013; **3**(1): 14–23

On page 15, we made the following material error: “*n* = 2199” should be “*n* = 21,991.”

We regret this error. It has no consequences for the reported analyses and conclusions.
